# Calpains play an essential role in mechanical ventilation-induced diaphragmatic weakness and mitochondrial dysfunction

**DOI:** 10.1016/j.redox.2020.101802

**Published:** 2020-11-25

**Authors:** Hayden W. Hyatt, Mustafa Ozdemir, Toshinori Yoshihara, Branden L. Nguyen, Rafael Deminice, Scott K. Powers

**Affiliations:** a Department of Applied Physiology and Kinesiology, University of Florida, Gainesville, FL, USA; b Department of Exercise and Sport Sciences, Hacettepe University, Ankara, Turkey; c Department of Exercise Physiology, Juntendo University, Tokyo, Japan; d Department of Physical Education, State University of Londrina, Londrina, Brazil

**Keywords:** Calpain, Muscle atrophy, Oxidative stress, Calpastatin, Muscle wasting, Muscle disuse

## Abstract

Mechanical ventilation (MV) is a life-saving intervention for many critically ill patients. Unfortunately, an unintended consequence of prolonged MV is the rapid development of diaphragmatic atrophy and contractile dysfunction, known as ventilator-induced diaphragm dysfunction (VIDD). Although the mechanism(s) responsible for VIDD are not fully understood, abundant evidence reveals that oxidative stress leading to the activation of the major proteolytic systems (i.e., autophagy, ubiquitin-proteasome, caspase, and calpain) plays a dominant role. Of the proteolytic systems involved in VIDD, calpain has received limited experimental attention due to the longstanding dogma that calpain plays a minor role in inactivity-induced muscle atrophy. Guided by preliminary experiments, we tested the hypothesis that activation of calpains play an essential role in MV-induced oxidative stress and the development of VIDD. This premise was rigorously tested by transgene overexpression of calpastatin, an endogenous inhibitor of calpains. Animals with/without transfection of the calpastatin gene in diaphragm muscle fibers were exposed to 12 h of MV. Results confirmed that overexpression of calpastatin barred MV-induced activation of calpain in diaphragm fibers. Importantly, deterrence of calpain activation protected the diaphragm against MV-induced oxidative stress, fiber atrophy, and contractile dysfunction. Moreover, prevention of calpain activation in the diaphragm forstalled MV-induced mitochondrial dysfunction and prevented MV-induced activation of caspase-3 along with the transcription of muscle specific E3 ligases. Collectively, these results support the hypothesis that calpain activation plays an essential role in the early development of VIDD. Further, these findings provide the first direct evidence that calpain plays an important function in inactivity-induced mitochondrial dysfunction and oxidative stress in skeletal muscle fibers.

## Highlights

• Inhibiting calpains during mechanical ventilation protects the diaphragm.
• Calpains play an important role in muscle atrophy and contractile dysfunction.
• Calpain inhibition during mechanical ventilation prevents mitochondrial dysfunction.
• Calpain-cleaved molecules may play important signaling roles.
• Calpain activation cross-talks with other proteolytic systems.

## Graphical abstract

**Figure:**
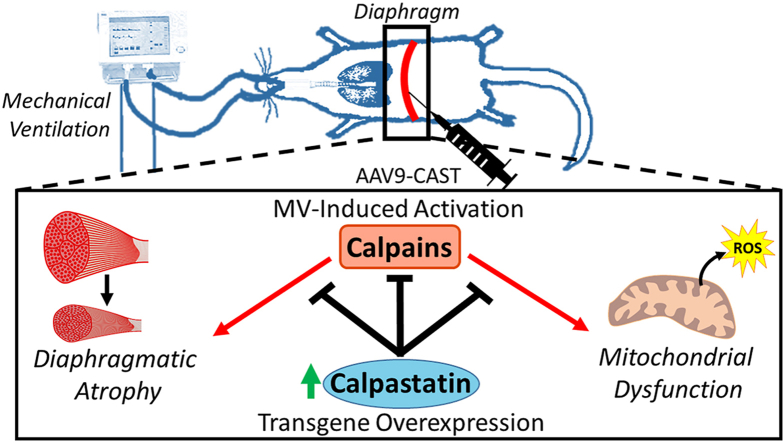

## 1 Introduction

Mechanical ventilation (MV) is used annually in >15 million patients worldwide to support the respiratory system during surgery and critical illness [1]. Although MV is often a life-saving intervention, prolonged MV results in the rapid development of inspiratory muscle weakness due to diaphragm atrophy and contractile dysfunction, collectively termed ventilator-induced diaphragm dysfunction (VIDD) [[2], [3], [4]]. VIDD is clinically important because diaphragmatic weakness is a major contributor to the inability to wean patients from the ventilator [[5], [6], [7]]. Difficulties in weaning patients from the ventilator are common with reports showing that ~40–70% of MV patients fail to wean from the ventilator during their first weaning attempt [8,9]. Problematic weaning results in extended hospitalization along with large increases in both ventilator-associated complications (e.g., infections) and patient mortality [8,10,11]. Unfortunately, the mechanism(s) responsible for VIDD remain poorly understood and thus, no clinical treatment exists. Understanding the cellular events that promote VIDD is essential to identify molecular targets for therapeutic intervention to prevent MV-induced diaphragmatic weakness.

While many unanswered questions remain about the pathogenesis of VIDD, progress continues toward understanding key cellular events leading to VIDD. For example, it is established that VIDD results from both accelerated proteolysis and depressed protein synthesis with proteolysis playing a dominant role [12]. Further, studies reveal that increased production of mitochondrial reactive oxygen species (ROS) is required for MV-induced protease activation in the diaphragm [13,14]. In this regard, all four major proteolytic systems (i.e., ubiquitin-proteasome, autophagy, caspase-3, and calpain) are active in the diaphragm during prolonged MV [[15], [16], [17], [18], [19]]. Nonetheless, recent evidence has highlighted the role of calpain-mediated pathogenesis in numerous tissues [[20], [21], [22]]. To date, only two reports have evaluated the impact of calpain activation on MV-induced diaphragmatic atrophy. Both studies concluded that pharmacological inhibition of calpains protects against VIDD [23,24]. These results challenge the long-standing dogma that calpains play a minor role in VIDD and other kinds of inactivity-induced muscle atrophy (e.g., prolonged bed rest). However, both studies suffer from the pitfall of utilizing calpain inhibitors with off-target effects (e.g., inhibition of cathepsins); hence, this shortcoming prohibits a clear interpretation of the data. Therefore, additional experiments that inhibit calpain exclusively are essential to establish the role of calpains in the occurrence of VIDD, and this forms the basis for the current experiments.

To determine the role that calpains play in the development of VIDD, we used a well-established animal model of MV and transfected the diaphragm with a recombinant adeno-associated viral vector (i.e., rAAV9) to overexpress the calpastatin (CAST) transgene, an endogenous inhibitor of calpain, to selectively inhibit calpain activation. Guided by our preliminary data, we tested the hypothesis that calpain activation plays an essential role in MV-induced oxidative stress and the consequential development of VIDD.

## 2 Methods

### 2.1 Animals and experimental design

Young adult (~4–6 months old) female Sprague-Dawley rats were randomly assigned to one of four experimental groups: 1) acutely anesthetized control animals (CON, n = 10); 2) animals receiving 12 h of mechanical ventilation (MV, n = 10); 3) animals with diaphragmatic overexpression of CAST that were acutely anesthetized (CON-CAST, n = 12); and 4) animals with diaphragmatic overexpression of CAST receiving 12 h of mechanical ventilation (MV-CAST, n = 12). Prior to experiments, all animals were housed in a 12:12 h light cycle with food and water provided *ad libitum*. The Institutional Animal Care and Use Committee of the University of Florida approved these experiments.

### 2.2 Experimental protocol

*Surgical protocol for AAV-CAST administration*- In order to determine the effects of calpain on diaphragm function, adeno-associated virus (AAV) vector containing CAST was delivered directly to the diaphragm via intramuscular injection as described previously [25]. Briefly, all animals were anesthetized and a laparotomy was performed followed by micro-injections in the diaphragm with either sterile saline (CON and MV) or AAV-9-CAST (CON-CAST and MV-CAST). This method of gene transfer effectively delivers gene transfer to the diaphragm and does not result in adverse side effects [25]. AAV vector containing CAST was purchased from Vector Biolabs (Malvern, PA, #AAV-204134) under control of the CMV promoter sequence. Animals were provided buprenorphrine (0.1 mg/kg) prior to awakening and every 12 h for 72 h following the surgery. Animals were allowed to recover for four weeks prior to sacrifice.

*Acutely anesthetized control animals-* Animals assigned to control groups were acutely anesthetized by IP injection of sodium pentobarbital (60 mg/kg body weight). After reaching a surgical plane of anesthesia, the heart was removed and costal diaphragm was collected for subsequent analyses. Animals in the acutely anesthetized control groups were sacrificed at the same time-point to animals in MV groups.

*Mechanical ventilation*- MV animals were tracheostomized and received mechanical ventilation for 12 h. Briefly, animals receiving MV were anesthetized with an IP injection of sodium pentobarbital (60 mg/kg body weight) and placed on a pressure-controlled ventilator (Servo Ventilator 300; Siemens, Munich, Germany). The ventilator was set at a respiratory rate of 80bpm and a positive end-expiratory pressure of 1cmH_2_O. The carotid artery was cannulated in order to measure blood pressure and the jugular vein was cannulated for continuous administration of anesthetic agent (sodium pentobarbital, 10 mg/kg/h). Arterial blood gasses were periodically measured (GEM Premier3000; Instrumentation Laboratory, Lexington, MA) in order to ensure that adequate PaO_2_ was maintained. Adjustments were made to ventilator settings controlling ventilation and oxygen fraction when needed. Specifically, PaO_2_ was maintained >60 mmHg and PaCO_2_ was maintained below 40 mmHg. Body temperature was maintained at 37 °C by a circulating water blanket and continuous care was provided to lubricate eyes, express bladder, and remove airway mucus throughout the 12-h period of MV. Glycopyrrolate (0.02 mg/kg) was administered intramuscularly every 2 h in order to reduce airway secretions. Upon completion of MV, costal diaphragm was quickly removed. Diaphragm tissue was separated for use of *in vitro* contractile measurements, mitochondrial isolation, and histochemical analyses. Remaining tissue was snap frozen in liquid nitrogen and stored at -80 °C for subsequent analyses.

### 2.3 Biochemical measures

*Mitochondrial isolation*- Mitochondria were isolated from fresh diaphragm tissue as described previously [26]. Briefly, costal diaphragm was placed in isolation buffer (100 mM KCl, 40 mM Tris HCl, 10 mM Tris base, 1 mM MgSO4, 0.1 mM EDTA, 0.2 mM ATP, and 0.15% (wt/vol) free fatty acid bovine serum albumin (BSA), pH 7.40) and minced with scissors on ice. Diaphragm was then homogenized with a polytron homogenizer for 7 s and trypsin (5 mg/g wet muscle) was added for 7 min. An equal volume of isolation buffer was added to the mixture and was centrifuged at 500×*g* for 10 min at 4 °C. The supernatant was removed and decanted through a layer of cheesecloth followed by centrifugation at 3500×*g* for 10 min. The supernatant was then removed and the pellet was suspended in isolation buffer without BSA. Following a final centrifugation at 3500×*g* for 10 min, the mitochondrial pellet was resuspended in 200 μL of a solution containing 220 mM mannitol, 70 mM sucrose, 10 mM Tris HCl, and 1 mM EGTA, pH 7.40.

*Mitochondrial respiration-* Oxygen consumption from freshly isolated mitochondria was measured polarographically in a respiration chamber (Hansatech Instruments, United Kingdom). Isolated mitochondria were placed in 1 mL of respiration buffer (100 mM KCL, 50 mM MOPS, 10 mM KH2PO4, 20 mM glucose, 10 mM MgCl2, 1 mM EGTA, and 0.2% fatty acid free BSA; pH = 7.0) at 37 °C and continually stirred. Oxygen consumption was measured during state 3 respiration with 2 mM pyruvate, 2 mM malate, and 0.25 mM ADP. State 4 respiration was measured following phosphorylation of ADP. Respiratory control ratio (RCR) was calculated as the oxygen consumption rate acquired during state 3 respiration divided by oxygen consumption rate during state 4 respiration.

*Mitochondrial oxidant emission*- Oxidant emission by isolated mitochondria were measured using Amplex™ UltraRed (Molecular Probes, Eugene, OR) (10 μM)/horseradish peroxidase (1 U/mL). Briefly, 10 μg of mitochondrial protein were loaded in wells containing Amplex™ UltraRed, 10 mM succinate, and horseradish peroxidase. Fluorescence was measured using a microplate reader set at 37 °C. Excitation and emission was set at 565/600 nm, respectively, and each measurement was made in triplicate. Values were calculated from standard curve utilizing H_2_O_2_. The rate of oxidant emission was expressed as H_2_O_2_ emissions in pmol·min^−^1 mg^−1^ protein. Mitochondrial protein concentrations were measured using the Bradford method.

*Western Blot-* Approximately 30 mg of costal diaphragm was homogenized in homogenization buffer (5 mM Tris-HCL, 5 mM EDTA; pH = 7.4) with a protease inhibitor cocktail (Sigma-Aldrich, St. Louis. MO) and centrifuged at 1500×*g* for 10 min at 4 °C. Diaphragmatic supernatant was collected and protein concentration was quantified by the method of Bradford (Sigma-Aldrich). Proteins were added to 2x Laemmli sample buffer (1610737, Bio Rad Hercules, CA) with 5% (w/vol) dithiothreitol, and boiled at 100 °C for 5 min. The supernatant fraction of proteins was separated via polyacrylamide gel electrophoresis and transferred to a polyvinylidene difluoride membrane. Membranes were probed for CAST (Abnova, Taipei, Taiwan, #H00000831-B01), αII-spectrin (Santa Cruz, Dallas, TX, sc-48382), 4-hydroxynonenal (Abcam, Cambridge, MA, ab46545), LC3 (Cell Signaling Technology, Danvers, MA, #2775), p-STAT3 (Cell Signaling Technology, #9145), STAT3-3 (Cell Signaling Technology, #4904), DRP1 (Santa Cruz, sc-271583), MFN1 (Abcam, ab104274), MFN2 (Abcam, ab56889), and PKC-δ (Abnova, #H00005580-M06). Revert total protein stain (LI-COR Biosciences, Lincoln, NE) was used to normalize for loading control in all western blots. For measurements of αII-spectrin degradation, the respective cleaved product was normalized to intact αII-spectrin. Additionally, proteins from isolated mitochondria were also separated and transferred to polyvinylidene difluoride membranes. Briefly, protease inhibitor cocktail was added to isolated mitochondria samples and subsequently were exposed to three freeze-thaw cycles in order to disrupt mitochondrial membranes. Mitochondrial fraction of proteins were probed for calpain1 (Capn1) (Cell Signaling Technology, #2556) and ATP5a1 (Santa Cruz, sc-136178). Membranes were imaged fluorescently and analyzed using the LI-COR Odyssey CLx Imaging System (LI-COR Biosciences, Lincoln, NE).

*Real-time polymerase chain reaction*- Total RNA was isolated from diaphragm with TRizol reagent (Life Technologies, Carlsbad, CA) according to the manufacturer's instructions. RNA content were evaluated by spectrophotometry and was subsequently reversed transcribed using SuperScript III First-Strand Synthesis System for RT-PCR (Life Technologies) and (dT)20 primers according to the protocol outlined by the manufacturer. Reactions for real-time PCR were prepared by adding 1 μL of cDNA to 25 μL to reaction mixture using Taqman chemistry and the ABI Prism 7000 Sequence Detection system (ABI, Foster City, CA). Atrogin-1/MAFbx (ThermoFisher Scientific, Rn00591730_m1), MuRF1 (Rn00590197_m1), and β-Glucuronidase (GUSB, (Rn00667869_m1) mRNA levels were quantified using relative quantification of gene expression via the comparative computed tomography method (ABI, User Bulletin #2). β-Glucuronidase was chosen as the reference gene based on previous work showing unchanged expression with our experimental manipulations [27].

### 2.4 Functional and histological measurements

*In vitro diaphragmatic contractile properties*- At the completion of experimental periods, a ~3 mm wide strip of costal diaphragm was placed in Krebs-Hensleit solution and equilibrated with 95% O_2_ – 5% CO_2_ gas. The strip was suspended vertically between Plexiglas clamps connected to an isometric force transducer (ModelFT-03, Grass Instruments, Quincy, MA) within a jacketed tissue bath. Muscle was electrically stimulated along its entire length using platinum wire electrodes (ModelFT-03, Grass Instruments, Quincy, MA). Optimum contractile length (Lo) was determined using supramaximal stimulation voltage (~150%). The force-frequency response was then measured by stimulating with 120-V pulses at 15–160 Hz. Maximal isometric twitch force was recorded at the 160 Hz frequency. Specific force was calculated by normalizing to physiological cross sectional area.

*Histological Measures-* Myofiber cross-sectional area (CSA) was determined using diaphragm frozen in OCT medium that was cut at 10 μm using a cryotome (Thermo HM 550 Cryostat, Thermo Scientific, Waltham, MA). Sections were stained for dystrophin (Thermo Scientific #RB-9024-R7), myosin heavy chain (MHC) I (Hybridoma Bank A4.840s IgM 1:15) and MHC type IIa (Hybridoma Bank SC-71c IgG 1:50) in order to determine CSA dependent on fiber type. Images were acquired via a monochrome camera attached to an inverted fluorescent microscope (Axiovert 200, Zeiss, Germany). CSA was determined using Scion software (NIH, Behesda, MD).

### 2.5 Statistical analysis

Comparisons between groups for each dependent variable were made by a one-way analysis of variance (ANOVA) and, when appropriate, a Tukey's HSD (honestly significant difference) test was performed *post-hoc*. For diaphragmatic specific force production, a one-way ANOVA was used to determine group differences at each stimulation frequency. Significance was established at p < 0.05. Data are expressed as mean ± SD.

## 3 Results

### 3.1 Calpastatin overexpression prevents calpain activation

**Fig. 1:**
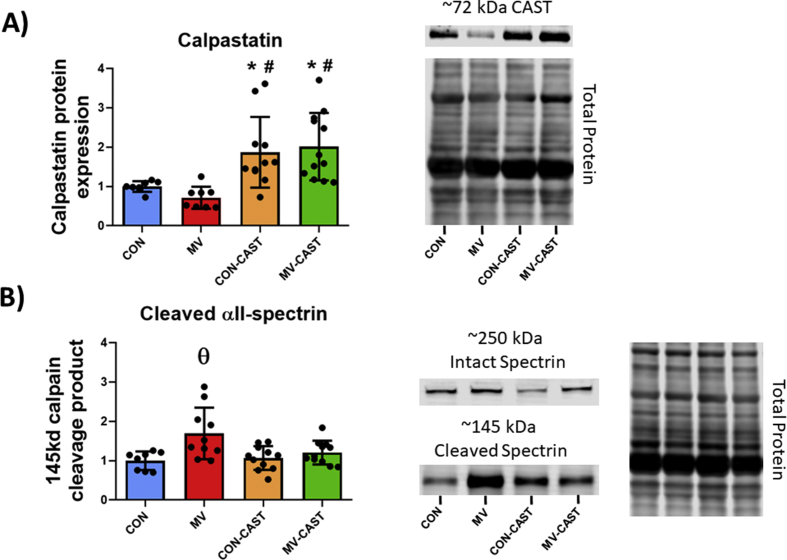
CAST overexpression prevents MV-induced activation of calpains. A) AAV9-CAST injections in diaphragm increase CAST expression in diaphragm fibers. B) Overexpression of CAST prevents MV-induced activation of calpains as determined by the calpain cleavage fragment of αII-spectrin. * = significantly different from CON, # = significantly different from MV, **θ** = significantly different from CON, CON-CAST, and MV-CAST.

To determine if the CAST transgene was overexpressed in the diaphragm, we measured CAST protein abundance in diaphragm muscle fibers via western blotting. Utilizing an antibody specific to the AAV9-CAST construct used in these experiments, our results indicate that CAST overexpression was achieved in CON-CAST and MV-CAST animals as indicated by CAST abundance being significantly greater (+75–100%) in CON-CAST and MV-CAST animals compared to both CON and MV animals (Fig. 1A).

To determine if CAST overexpression successfully prevented the MV-induced activation of calpains in the diaphragm, we measured the abundance of the 145 kDa calpain-specific αII-spectrin cleavage fragment. Specifically, this 145 kDa spectrin cleavage fragment has a relatively long half-life of ~4.2 h and is widely used as a biomarker of calpain activity *in vivo* [23,[28], [29], [30]]. Our results reveal that overexpression of CAST prevented MV-induced increases in calpain activity in the diaphragm as demonstrated by the significantly higher abundance of the 145 kDa αII-spectrin cleavage product in MV animals compared to all other groups (Fig. 1B). Collectively, our results demonstrate that CAST was successfully overexpressed and that CAST overexpression prevented MV-induced increases in calpain activity.

CAST overexpression does not impact diaphragm fiber CSA or contractile function in acutely anesthetized animals.

**Fig. 2:**
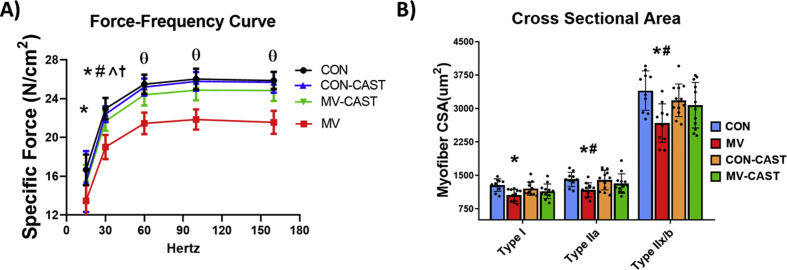
CAST overexpression protects the diaphragm against MV-induced contractile dysfunction and muscle atrophy. A) Force-frequency curve of diaphragm muscle. B) Cross-sectional area of diaphragm fibers. * = MV is significantly different from CON, # = MV is significantly different from CON-CAST, **^** = MV-CAST is significantly different from CON, † = Con is significantly different from MV-CAST θ = MV is significantly different from CON, CON-CAST, and MV-CAST.

Although the only known function of CAST is the inhibition of calpains [31,32], it is currently unknown if overexpression of CAST negatively impacts skeletal muscle fiber size or function. Therefore, we compared diaphragm fiber size and contractile function in acutely anesthetized animals overexpressing CAST (i.e., CON-CAST) with control animals. Importantly, compared to control, overexpression of CAST in diaphragm fibers did not affect contractile function (Fig. 2A), fiber size (Fig. 2B), or any other dependent variable measured within this study.

### 3.2 Inhibiting MV-induced calpain activation in the diaphragm protects against VIDD

To determine if prevention of MV-induced calpain activation in the diaphragm protects against VIDD, we measured diaphragm fiber CSA and muscle specific-force production. As expected, compared to control, our results demonstrate that diaphragm muscle fiber CSA and specific force production is decreased in MV animals. Importantly, prevention of MV-induced calpain activation in the diaphragm via CAST overexpression protected the diaphragm against both MV-induced atrophy and contractile dysfunction (Fig. 2A and B).

### 3.3 Prevention of MV-induced calpain activation in the diaphragm protects against ventilator-induced mitochondrial dysfunction and oxidative stress

**Fig. 3:**
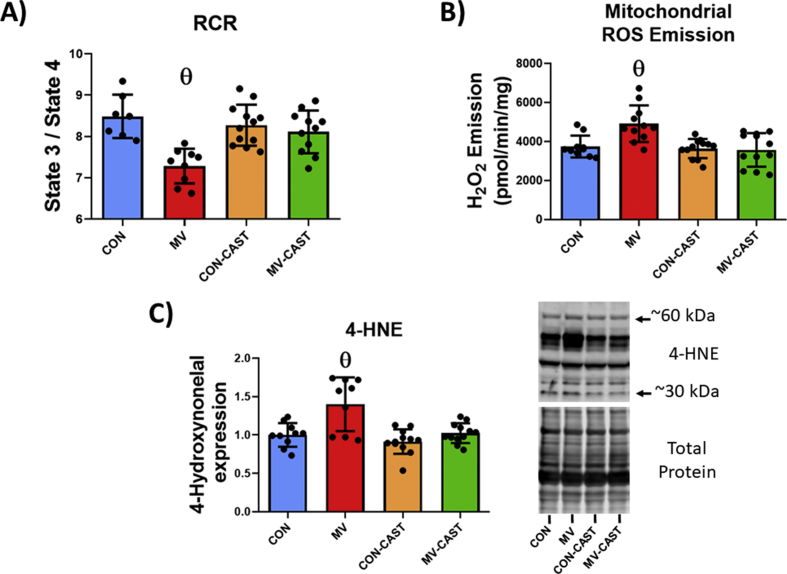
Preventing calpain activation protects against MV-induced mitochondrial dysfunction and oxidative stress. A) Respiratory control ratio of isolated mitochondria. B) ROS emission from isolated mitochondria. C) Marker of oxidative damage 4-HNE expression. **θ** = Significantly different from CON, CON-CAST, and MV-CAST.

Mitochondrial dysfunction and increased mitochondrial ROS emission in diaphragm fibers are hallmarks of VIDD and are a contributing cause of VIDD [13,33]. To determine if calpain activation plays a role in MV-induced mitochondrial dysfunction, we measured both mitochondrial respiration and mitochondrial ROS emission from mitochondria isolated from diaphragm muscle. Prevention of MV-induced calpain activation via transgenic overexpression of CAST successfully averted MV-induced mitochondrial dysfunction. Indeed, CAST overexpression prevented MV-induced mitochondrial uncoupling (i.e., decreases in RCR) and prevented MV-induced increases in mitochondrial ROS emission (Fig. 3A and B). Furthermore, inhibiting calpains prevented MV-induced increases in diaphragmatic levels of 4-HNE, a biomarker of oxidative stress (Fig. 3C). Collectively, these results reveal that calpains play an integral role in the promotion of MV-induced mitochondrial dysfunction and increased mitochondrial ROS emission in diaphragm muscle fibers.

### 3.4 MV-induced activation of calpain in the diaphragm increases bioactive proteins associated with mitochondrial dysfunction

**Fig. 4:**
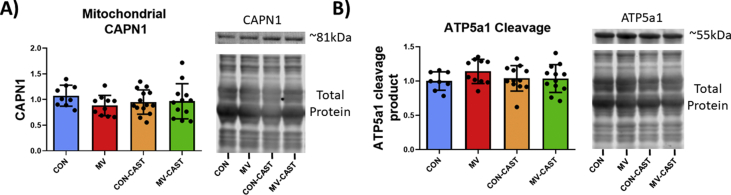
Preventing calpain activation during MV does not alter calpain abundance or calpain cleavage of ATP5a1 within isolated mitochondria. A) Mitochondrial content of CAPN1 is not increased with MV B) Mitochondrial ATP5a1 is not cleaved by calpains during MV. No significant differences detected.

**Fig. 5:**
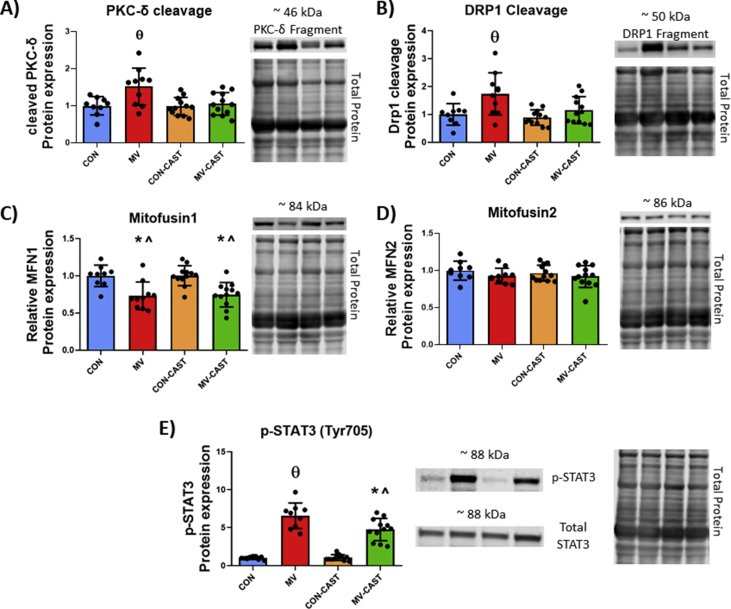
MV-induced calpain activation results in altered mitochondrial regulatory proteins. A) Calpain-cleaved PKC-δ cleavage fragment is increased with MV. B) Calpain-cleaved DRP1 cleavage fragment is increased with MV. C) MFN1 is decreased with MV. D) MFN2 is unchanged with prolonged MV. E) Phosphorylation of STAT3 is increased with MV and blunted with CAST overexpression. * = significantly different from CON, **^** = significantly different from CON-CAST, **θ** = significantly different from CON, CON-CAST, and MV-CAST.

Calpains are capable of cleaving substrate proteins into peptide/small protein fragments that have altered biological function relative to the intact protein [29]. To determine potential mechanisms responsible for the link between MV-induced calpain activity and mitochondrial dysfunction in diaphragm fibers, we measured the abundance of intact and catalyzed protein fragments that are confirmed to promote mitochondrial dysfunction. For instance, a previous report concludes that calpain1 can accumulate in mitochondria and promote mitochondrial dysfunction via cleavage of ATP5a1 [22]. In the current study, we observed no differences in the mitochondrial content of calpain1 or ATP5a1 cleavage fragment measured from isolated mitochondria (Fig. 4A and B). In contrast, we discovered an increased abundance of protein cleavage fragments in the cytosol of diaphragm fibers that have been implicated in mitochondrial dysfunction. Specifically, our findings reveal that cleavage fragments of both PKC-δ and DRP1 were elevated in diaphragm homogenate from MV group animals (Fig. 5A and B). In this regard, calpain-mediated cleavage of PKC-δ results in a catalytically active fragment that appears at a molecular weight of ~45 kDa [34,35]. In addition, calpains cleave DRP1 generating a DRP1 cleavage fragment that appears at ~50 kDa [36]. CAST overexpression prevented the MV-induced increase in PKC-δ and DRP1 cleavage fragments.

Prolonged MV also resulted in a decreased abundance of the mitochondrial fusion protein mitofusin 1 (MFN1); however, calpastatin overexpression did not rescue MV-induced decreases in MFN1 abundance (Fig. 5C). In contrast, no differences were detected for MFN2 protein abundance between the experimental groups (Fig. 5D). Lastly, phosphorylation of STAT3 at Tyr705 has been reported to promote mitochondrial dysfunction [37]. Our findings show that CAST overexpression attenuated MV-induced phosphorylation of STAT3 in the diaphragm (Fig. 5E).

### 3.5 MV-induced calpain activation promotes activation of caspase-3 and transcription of key ubiquitin-proteasome system genes in the diaphragm

**Fig. 6:**
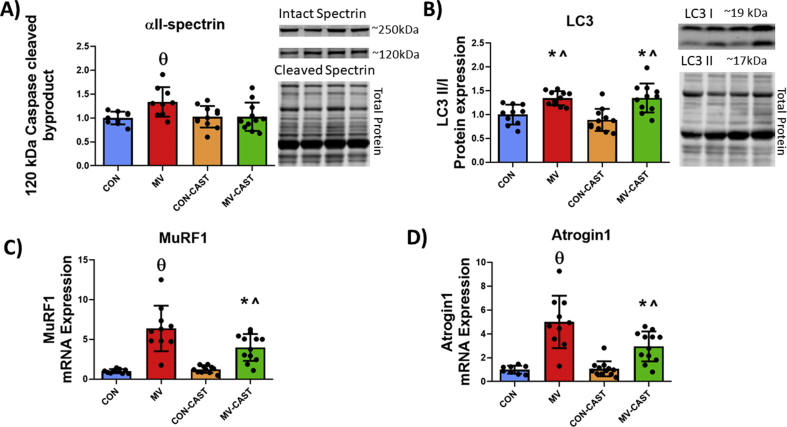
Inhibiting calpains with CAST overexpression suggests cross-talk of calpains with other proteolytic systems during MV. A) Caspase-cleaved spectrin indicating activity of caspase system. B) LC3 II/I protein expression. C) MuRF1 mRNA levels D) Atrogin1 mRNA levels. * = significantly different from CON, **^** = significantly different from CON-CAST, **θ** = significantly different from CON, CON-CAST, and MV-CAST.

To determine if MV-induced calpain activation promotes activation of other key proteolytic systems in the diaphragm, we measured biomarkers of the activation of caspase-3, autophagy, and ubiquitin-proteasome system. Preventing the activation of calpains during MV attenuated caspase-3 activation as demonstrated by increased abundance of the 120 kDa caspase-3 cleavage fragment of αII-spectrin in MV group animals compared to all other groups (Fig. 6A). In addition, CAST overexpression ameliorated MV-induced increases in mRNA levels of the muscle specific E3 ligases MuRF1 and Atrogin1 (Fig. 6C and D). In contrast, preventing calpain activation did not alter the MV-induced increase in the abundance of LC3II/I ratio, a marker of autophagosome formation (Fig. 6B). Collectively, these results demonstrate cross-talk between calpain activation and activation the caspase-3 and ubiquitin-proteasome systems.

## 4 Discussion

These experiments provide important insight into the role that calpains play in VIDD. Indeed, our results provide the first robust proof that calpains directly participate in promoting VIDD and that prevention of MV-induced calpain activation protects the diaphragm against both fiber atrophy and contractile dysfunction. Furthermore, our findings offer the first evidence that prevention of calpain activation protects against VIDD by avoiding calpain-mediated mitochondrial dysfunction and accelerated ROS production; together, these findings corroborate that calpains are a required up-stream signaling effector contributing to VIDD. Although the precise details of how calpain activation orchestrates the signaling steps leading to VIDD remain unclear, our results identify several possible scenarios of how calpains interact with mitochondria and other proteases to promote the rapid development of VIDD. A critique of our experimental approach and a detailed discussion of our results follows.

### 4.1 Critique of experimental approach

Due to the invasive nature of acquiring human diaphragm muscle biopsies, animal models are required to investigate the mechanism(s) responsible for VIDD. The current experiments used a well-established animal model to test the hypothesis that activation of calpain plays an essential role in MV-induced oxidative stress and the development of VIDD. We selected the rat as an experimental model because rat and human diaphragm muscles share similar anatomical features, functional characteristics, fiber type composition, and exhibit a parallel time course in the development of VIDD [[2], [3], [4],[38], [39], [40]]. Female rats were chosen for study because they maintain a stable body weight from 4 to 6 months of age, and no gender differences exist between male and female rats in their development of VIDD [41,42].

Two prior studies have reported that pharmacological inhibition of calpain activation protects against VIDD [23,24]. Unfortunately, a clear interpretation of these studies is not possible due to off-target effects of the calpain inhibitors employed (e.g., inhibition of cathepsins); therefore, studies that avoid this experimental pitfall are essential to determine the direct effects of calpain activation on VIDD. The current study avoids the experimental pitfall of pharmacological off-target effects by inhibiting calpain activation via transgene overexpression of CAST in the diaphragm. Specifically, we used AAV-9 transduction of gene transfer to diaphragm muscle to overexpress CAST in diaphragm fibers; this method has been shown to successfully overexpress target genes without adverse effects on muscle fibers [25]. Furthermore, expression of empty-AAV9 viral vector does not alter muscle force production or fiber CSA [25]. We selected CAST to inhibit calpain activation because CAST is an endogenously expressed protein, and the only known function of CAST is the inhibition of calpains [43]. Importantly, our experiments confirm that overexpression of CAST in the diaphragm does not alter diaphragm fiber size or contractile function.

In regard to the calpain isoforms responsible for VIDD, calpain 1, calpain 2, and calpain 3 are the predominant isoforms expressed in skeletal muscle [29]. However, CAST does not inhibit calpain 3 activity [44]; therefore, our findings that CAST overexpression protects the diaphragm against VIDD indicate that calpain 1 and/or calpain 2 isoforms are the primary calpain isoforms responsible for the occurrence of VIDD.

### 4.2 Calpains play an essential role in VIDD

VIDD occurs within the first 12–18 h of prolonged MV in both humans and animals as evidenced by a 15–20% reduction in both diaphragm fiber size and specific force production [[2], [3], [4]]. While previous studies have demonstrated that this rapid rate of MV-induced diaphragmatic muscle atrophy is driven by accelerated proteolysis [42,45,46], the current experiments provide the first robust evidence that activation of calpain is essential for the occurrence of VIDD. Indeed, because the only known function of CAST is to inhibit calpain, our results confirm that prevention of MV-induced activation of calpain protects the diaphragm against MV-induced reductions in both fiber CSA and contractile forces. These important findings identify calpain as a potential therapeutic target to protect against both VIDD and problems in weaning patients from the ventilator.

Our findings are in direct contrast with the dogma that calpains do not play a significant role in promoting disuse muscle atrophy. Indeed, historically, it has been believed that the primary function of calpains is to assist other proteolytic systems in protein turnover, and therefore, active calpains play a minor role in inactivity-induced muscle atrophy [47]. Our findings challenge this doctrine and reveal that calpains play a pervasive role in coordinating VIDD. Although the precise mechanisms behind calpain-mediated muscle atrophy remain unclear, our results provide evidence of several potential processes by which calpains contribute to MV-induced diaphragmatic atrophy.

### 4.3 Calpains promote MV-induced mitochondrial dysfunction in diaphragm fibers

Mitochondrial dysfunction in diaphragm muscle fibers is a hallmark of prolonged MV [13,33]. This mitochondrial dysfunction is associated with increased mitochondrial ROS emission that is concomitant with oxidation of both proteins and lipids in diaphragm fibers [13,33,48]. The importance of mitochondrial dysfunction during VIDD is confirmed by studies showing that mitochondrial-targeted antioxidant peptides prevent MV-induced mitochondrial dysfunction and protect against VIDD [13,14]. In the current study, we demonstrate that prevention of calpain activation protects diaphragm fibers against both MV-induced uncoupling of mitochondrial respiration and increased ROS emission. To our knowledge, this is the first direct evidence that inhibition of calpain activation averts MV-induced oxidative damage to diaphragmatic proteins. Intriguingly, previous reports have shown that preventing MV-induced increases in mitochondrial ROS emission prevents activation of calpains in diaphragm fibers [13,14]. Collectively, these findings suggest that cross-talk exists between calpain and increased mitochondrial dysfunction/ROS emission. While the process linking mitochondrial dysfunction to calpain activation is likely increased ROS emission from mitochondria [13,49], the mechanistic link explaining the effects of calpain activation on mitochondrial function remains unknown.

The function of calpains in skeletal muscle is canonically viewed as supporting myofibrillar protein turnover by releasing sarcomeric proteins for degradation by other proteases (e.g., ubiquitin-proteasome system). In contrast to this view, evidence from previous studies investigating the effect of active calpain on non-muscle cell types suggests that calpains play several roles as signaling effectors in cellular proteolysis [29]. Indeed, calpains are unique proteases capable of contributing to cell signaling via cleavage of target proteins into biologically active fragments. It follows that these biologically active protein fragments have the potential to serve as signaling molecules in diaphragm fibers during MV.

To identify potential calpain-signaling events contributing to mitochondrial dysfunction, we explored three calpain-mediated events that are reported to promote mitochondrial dysfunction. First, evidence indicates that calpains can accumulate in mitochondria resulting in cleavage of the ATP synthase subunit, ATP5a1, ensuing in mitochondrial dysfunction [22]. Nonetheless, our results indicate that calpain abundance does not increase in diaphragm mitochondria during MV. In contrast, our findings reveal that calpain activation during MV results in the accumulation of cleavage fragments of both PKC-δ and DRP1 in the soluble fraction of diaphragm homogenate. This is potentially important because calpain-induced cleavage of PKC-δ results in a catalytically active protein with higher PKC-δ enzymatic activity [34,35]. While the role of PKC-δ in skeletal muscle is not well defined, evidence from other cell types confirms that PKC-δ promotes mitochondrial dysfunction by increasing mitochondrial ROS production, mitochondrial fission, mitochondrial permeable transition pore opening, and cytochrome C release [[50], [51], [52], [53], [54]]. For example, the cleaved PKC-δ fragment has been reported to interact with the mitochondrial fission protein DRP1 and translocate to mitochondria resulting in increased mitochondrial fission [53,54]. Furthermore, cleaved PKC-δ participates in the phosphorylation of STAT3 at Tyr705 [55]. Phosphorylated STAT3 translocates into the mitochondria to a site within complex I and is associated with mitochondrial dysfunction in diaphragm fibers during MV [37]. Our results disclose that, compared to MV animals, MV-induced phosphorylation of STAT3 at Tyr705 in diaphragm fibers was ameliorated in MV-CAST animals. Therefore, calpain-mediated increases in the abundance of a catalytically active PKC-δ fragment is a potential candidate to contribute to MV-induced mitochondrial dysfunction and the downstream effects.

Our results also provide evidence of accumulation of a DRP1 cleavage fragment in diaphragm fibers during MV. Although the physiological relevance of this DRP1 cleavage protein remains unclear, recent evidence indicates that cleavage of DRP1 by calpains contributes to mitochondrial dysfunction in Alzheimer's disease [36]. In this regard, disruptions in the balance of mitochondrial fission and fusion have been shown to result in mitochondrial dysfunction and promote muscle atrophy [56,57]. Specifically, new evidence indicates that DRP1 deficiency results in mitochondrial dysfunction resulting in skeletal muscle wasting and weakness [57]. In reference to prolonged MV and mitochondrial dysfunction, as few as 6 h of MV results in diaphragm mitochondria that are morphologically deranged and dysfunctional [58]. It follows that calpain-mediated cleavage of DRP1 is a potential mechanism by which calpain activation induces mitochondrial dysfunction during MV. This, coupled with our findings of decreased MFN1 abundance, suggest an imbalance exists between the processes of mitochondrial fission and fusion which may underlie the altered mitochondrial morphology associated with prolonged MV. Nonetheless, additional research is required to experimentally confirm cause and effect of calpain-cleaved DRP1 and PKC-δ fragments in MV-induced diaphragmatic atrophy.

### 4.4 Prolonged MV promotes cross-talk between calpain and other proteases in diaphragm fibers

After discovering that MV-induced calpain activation is required to promote oxidative stress in the diaphragm, we then conjectured that calpain activation is a required upstream signal that promotes activation of caspase-3, ubiquitin-proteasome system, and autophagy in the diaphragm. This postulate was fostered by evidence that oxidative stress is required for MV-induced activation of key proteolytic systems in diaphragm fibers [13,48,59]. Further, both *in vitro* and *in vivo* experiments reveal that active calpains stimulate the activation of caspase-3 [23,60]. Our results confirm that prevention of MV-mediated activation of calpain averted the MV-induced activation of caspase-3 and the increased transcription of muscle specific E3-ligases (i.e., Atrogin1 and MuRF1) in diaphragm fibers. In contrast, prevention of calpain activation in diaphragm fibers did not prevent the MV-induced increase in a biomarker of autophagosome formation (i.e., LC3II/I ratio). Note, however, that the measurement of LC3II/I as a biomarker of autophagic flux has limitations and therefore, we cannot exclude the possibility that active calpain influences autophagy in diaphragm fibers [61].

Together, these findings provide the first evidence that overexpression of a CAST transgene in diaphragm fibers prevents MV-induced calpain activation and avoids MV-mediated increases in the transcription of muscle specific E3 ligases, as well as the activation of caspase-3. These results confirm that biological crosstalk exists between calpains and both the ubiquitin-proteasome system and caspase-3 in the diaphragm during prolonged MV. While our data corroborate a connection between calpain and activation of other proteolytic systems, our results do not disclose the mechanism(s) responsible for the calpain driven activation of caspase-3 and expression of muscle specific E3 ligases. Nonetheless, several possibilities exist for this connection. First, active calpain can cleave procaspase-12 into its active form, caspase-12 [60]. Active caspase-12 can then indirectly activate caspase-3 [62]. Alternatively, active calpain can act on pro-apoptotic proteins such as Bid and Bax [63]. Following activation, these pro-apoptotic factors often form a pore in the outer membrane of mitochondria resulting in the release of cytochrome C into the cytosol [64]; cytosolic cytochrome C can then activate caspase-3 via formation of the apoptosome followed by activation of caspase-9 [65]. Hence, a solid theoretical basis exists to explain the cell-signaling cross-talk between calpain and caspase-3 in muscle fibers.

Caspase-3 activation resulting in apoptotic signaling may act as another mechanism of calpain-induced muscle wasting. For instance, myonuclear apoptosis is increased with prolonged MV in a caspase-dependent manner [66]. In theory, myonuclear apoptosis can contribute to disuse muscle atrophy by reducing the transcriptional activity that precedes protein synthesis [67]. In this regard, caspase-3 knockout mice are resistant to denervation-induced myonuclear apoptosis and muscle atrophy [68]. Therefore, calpain-induced activation of caspse-3 may initiate apoptotic signaling and contribute to diaphragmatic atrophy during MV.

In addition to the direct effect of calpain on caspase-3 activity, the fact that calpain promotes oxidative stress in the diaphragm during prolonged MV provides another potential mechanism by which active calpain promotes accelerated proteolysis. For example, oxidative modification of skeletal muscle proteins increases the susceptibility of these proteins to calpain and caspase-3 degradation [49]. Indeed, oxidation of myofibrillar proteins increases their susceptibility to proteolysis due to unfolding of the molecule [69]. More specifically, oxidation of muscle proteins leads to the modification of the secondary or tertiary structure of the protein molecule so that shielded peptide bonds are exposed and susceptible to enzymatic hydrolysis [[69], [70], [71]].

## 5 Summary and conclusions

Although MV is a life-saving intervention for many critically ill patients, an unintended consequence of prolonged MV is the development of diaphragmatic weakness (i.e., VIDD). VIDD is a clinically important because diaphragmatic weakness is a major risk factor for problems in weaning patients from the ventilator [6,[72], [73], [74]]. Currently, no clinical treatment exists and a detailed knowledge about the signaling events that promote VIDD is essential to develop a pharmacological therapy to prevent MV-induced diaphragmatic weakness. Notably, the current investigation identifies calpains as a potential therapeutic target to combat VIDD as prevention of MV-induced activation of calpain in the diaphragm guards against VIDD. These results challenge the long-standing dogma that calpains play a minor role in VIDD and muscle atrophy. Moreover, our data are the first to reveal that prevention of MV-induced calpain activation protects diaphragm muscle fibers from mitochondrial damage, oxidative stress, and activation of caspase-3 as well as the expression of muscle specific E3 ligases during prolonged MV. Collectively, these findings highlight the important role that calpains play in the development of VIDD and provide insight into the mechanistic links between calpain and accelerated proteolysis in the diaphragm during prolonged MV.

## Funding

This work was supported by a grant from the 10.13039/100000002National Institutes of Health (10.13039/100000002NIH R21 AR073956) awarded to SKP.

## Declaration of competing interest

None.
